# Parental awareness and practice on malocclusion in Ahmedabad, Gujarat, India: A questionnaire Survey

**DOI:** 10.6026/973206300214116

**Published:** 2025-11-15

**Authors:** Narayan Kulkarni, Ipsit Trivedi, Harsh S Modi, Nirali Shah, Lay Doshi

**Affiliations:** 1 Department of Orthodontics and Dentofacial Orthopedics, Goenka research institute of Dental science (GRIDS), Gandhinagar, Gujarat, India

**Keywords:** Children, orthodontics, parental knowledge

## Abstract

Malocclusion in children often remains unnoticed or underestimated by parents, despite its significant impact on oral function and psychosocial well-being. Therefore, it is of interest to assess parental awareness and practices regarding malocclusion and orthodontic care among 200 parents in Ahmedabad, Gujarat, using a validated bilingual questionnaire. Data revealed that while 75.5% of parents were aware of malocclusion, only 30% knew about orthodontic appliances beyond fixed metal braces and male parents demonstrated significantly higher awareness levels. Most parents (86%) expressed readiness to persuade their child for treatment based on professional advice; yet socioeconomic factors influenced treatment preferences. Thus, that despite moderate awareness, there is a pressing need for targeted public health programs and educational initiatives to enhance parental knowledge and encourage timely orthodontic intervention for children.

## Background:

Malocclusion refers to the improper alignment or positioning of teeth and dental arches, leading to deviations from the normal occlusal relationship [1]. It is recognized as one of the most prevalent dental problems worldwide and ranks third after dental caries and periodontal diseases [2]. Malocclusion not only affects oral function but also has aesthetic, social and psychological implications that can influence an individual's self-esteem and overall quality of life [3, 4-5]. In growing children and adolescents, such irregularities can interfere with normal jaw development, speech and mastication, making early detection and management crucial [6, 7]. Orthodontic treatment aims to correct these discrepancies, thereby improving facial harmony, dental function and psychological well-being [8]. Children and adolescents often become self-conscious about their dental appearance as they grow older, yet the decision to seek orthodontic treatment typically depends on their parents, who play a decisive role due to financial, social and health-related considerations [9, 10, 11-12]. The level of parental awareness, perception and attitude toward malocclusion directly influences whether children receive timely orthodontic evaluation and intervention [13]. Lack of awareness or misconceptions about the importance of early orthodontic assessment can delay treatment, potentially worsening dental and skeletal discrepancies over time [14, 15-16]. Therefore, understanding and enhancing parental knowledge regarding malocclusion and orthodontic care is essential to promote preventive measures, encourage early treatment and achieve better oral health outcomes for children.

## Methodology:

This was a cross-sectional questionnaire-based study designed to assess parental awareness and practices regarding malocclusion. The sample size was calculated using Open Epi Stat Calc version 3.01, with a confidence level of 95%, power of 80 and an anticipated frequency of 50%. Based on these parameters, a sample of 194 participants was determined [17]. The data collection was carried out over a period of three months (From October to December 2024). A total of 194 completed questionnaires comprising of 97 male and 97 female parents were collected and included in the final analysis. Convenient random sampling was used to reach parents from diverse socio-economic backgrounds across multiple locations in West Ahmedabad, enhancing representativeness. Parents or guardians of children aged 7 to 18 years, residing in West Ahmedabad and who provided signed informed consent were included. Individuals with formal dental or orthodontic qualifications, non-parent adults and residents outside West Ahmedabad, those related to dentists or orthodontists and those who did not provide consent or submitted incomplete or late questionnaires were excluded. Manual distribution of the questionnaire allowed researchers to reach a broader section of the community, including participants with limited access to digital resources, ensuring representation from varied socio-economic strata. A structured questionnaire was developed through literature review, expert consultation and adaptation of validated survey instruments. Initially consisting of 15 questions, it was refined to 10 close-ended questions (7 on awareness and 3 on practices) based on expert feedback (Figure 1 - see PDF). Content validity was reviewed by five orthodontic experts, leading to revisions in two questions for improved clarity. Internal consistency was measured using Cronbach's Alpha (0.85), indicating high reliability. The questionnaire was translated into English and Gujarati to improve comprehension among participants. A pilot study with 20 parents was conducted to assess clarity and repeatability of the language.

## Results:

The study assessed parental awareness and attitudes toward malocclusion and orthodontic treatments. Among the respondents, 76.5% were male and 23.5% were female. A majority of parents (59%) had daughters, while 41% had sons. Regarding awareness, 75.5% of parents were familiar with malocclusion, 61% recognized its hereditary nature and 72% understood that certain childhood habits could contribute to its development. Additionally, 71.5% acknowledged that orthodontic treatment differs for children and adults, while 79.5% were aware that early interceptive treatment can reduce the need for future orthodontic care. Furthermore, 70% recognized that timely intervention decreases the likelihood of surgical correction later in life. Only 30% were aware of orthodontic appliances beyond traditional fixed metal braces (Table 1 - see PDF). In terms of parental behavior, 62% regularly monitored their child's dental alignment. A significant majority (86%) expressed willingness to persuade their child to undergo treatment based on professional recommendations. 85.5% of parents favored public treatment centers and 83% were willing to endure extended waiting periods as required. Statistical analysis revealed significant gender-based differences in awareness and practices. Male parents demonstrated greater knowledge of malocclusion (p < 0.001), its hereditary nature (p = 0.001) and its association with childhood habits (p < 0.001). They were also more informed about the benefits of early interceptive treatment (p = 0.001) and its role in reducing the need for surgical intervention (p < 0.001). Additionally, male parents were more likely to monitor their child's teeth alignment (p = 0.002) and persuade them to seek treatment (p < 0.001). However, no significant gender differences were observed in the preference for public versus private treatment centers (p = 0.301) or willingness to wait for treatment at public facilities (p = 0.181) (Table 2 - see PDF).

## Discussion:

Malocclusion is one of the most prevalent dental issues in children and its early diagnosis and management play a crucial role in reducing future complications and improving oral health outcomes [18]. However, early orthodontic intervention largely depends on the awareness, attitude and decision-making capacity of parents [19]. In areas like West Ahmedabad, there has been limited focus on evaluating parental awareness and attitudes toward orthodontic care. Most existing literature has emphasized the clinical prevalence of malocclusion rather than the perceptions and behaviours of parents regarding orthodontic treatment. Therefore, this study was undertaken to bridge that gap and explore parental awareness, attitudes and practices, which are critical for guiding early orthodontic intervention and planning targeted educational programs [20]. To accurately assess the parental perspective, a structured and validated questionnaire was designed. The questionnaire development process involved a thorough literature review, consultations with orthodontic experts and adaptation of previously validated survey tools. Content validity was established through expert review and internal consistency was confirmed with a Cronbach's Alpha score of 0.85 [21]. The questionnaire was translated into English and Gujarati to accommodate the linguistic needs of the study population and tested through a pilot study for clarity and repeatability. This rigorous validation process ensured the relevance and reliability of the tool for assessing awareness and practices related to malocclusion and orthodontic care in a culturally diverse population. Our findings revealed that 75% of parents demonstrated adequate knowledge, while 51% showed a positive attitude toward orthodontic treatment. This aligns with studies conducted in similar urban populations, which also reported moderately high awareness and varying degrees of acceptance of orthodontic care [22]. This pattern has been seen in studies conducted in other regions and socioeconomic contexts [23]. Aesthetic concerns were the most frequently cited motivation for seeking treatment, which reflects the growing social importance of dental appearance. However, consistent with previous studies, lower and middle-income parents often preferred subsidized government dental services due to cost-related constraints, even when their children had clear treatment needs [24]. Maxillary canine impaction is a common dental anomaly affecting eruption beyond the age of 11-12 years, often leading to complications such as root resorption and aesthetic issues [25]. While this study provides valuable insights, several limitations must be acknowledged. The use of convenience sampling may affect the generalizability of the results to the broader population. The reliance on self-reported data introduces the potential for recall and social desirability biases. Furthermore, the study was geographically limited to West Ahmedabad, which restricts applicability to other regions. The absence of a clinical examination component also means that parental perceptions could not be directly correlated with actual orthodontic treatment needs. Despite careful translation of the questionnaire, language interpretation issues may have influenced the accuracy of responses. Additionally, external influences such as parental education level, previous dental experiences and accessibility to orthodontic services were not comprehensively evaluated. This study highlights the importance of enhancing parental awareness regarding malocclusion and the need for early orthodontic intervention. The findings suggest that educational efforts should focus on both improving knowledge and addressing psychosocial and economic barriers to treatment. Clinicians and public health policymakers should consider expanding access to orthodontic care by prioritizing not only clinical need but also the psychosocial well-being of the child and the socioeconomic background of the family. There is a pressing need to revise existing public health policies to accommodate families from lower socioeconomic backgrounds by increasing the availability of subsidized orthodontic services. Clinicians should also play an active role in community outreach and parental counselling to promote timely treatment, especially in underrepresented populations.

## Conclusion:

Parents generally exhibited moderate to high levels of knowledge and appropriate practices regarding malocclusion and orthodontic treatment for their children. Within the study population, female participants demonstrated a greater emphasis on esthetic concerns, whereas male participants were more influenced by financial considerations. Additionally, parents from higher socioeconomic backgrounds exhibited greater proactivity in seeking orthodontic care. Thus, we show the need for public health policies that emphasizes on provision of financial subsidy on orthodontic care and services.
